# Supplementary material for: Energy transformation cost for the Japanese mid-century strategy
Source: Nat Commun. 2019 Oct 18;10:4737. doi: 10.1038/s41467-019-12730-4 (PMC6800421; doi:10.1038/s41467-019-12730-4)
Supplement: Supplementary file 2 — Supplementary Information [file 41467_2019_12730_MOESM2_ESM.pdf]

SUPPLEMENATRY INFORMATION for

Energy Transformation Cost for the Japanese Mid-century Strategy

Fujimori et al.

1. Supplementary figures

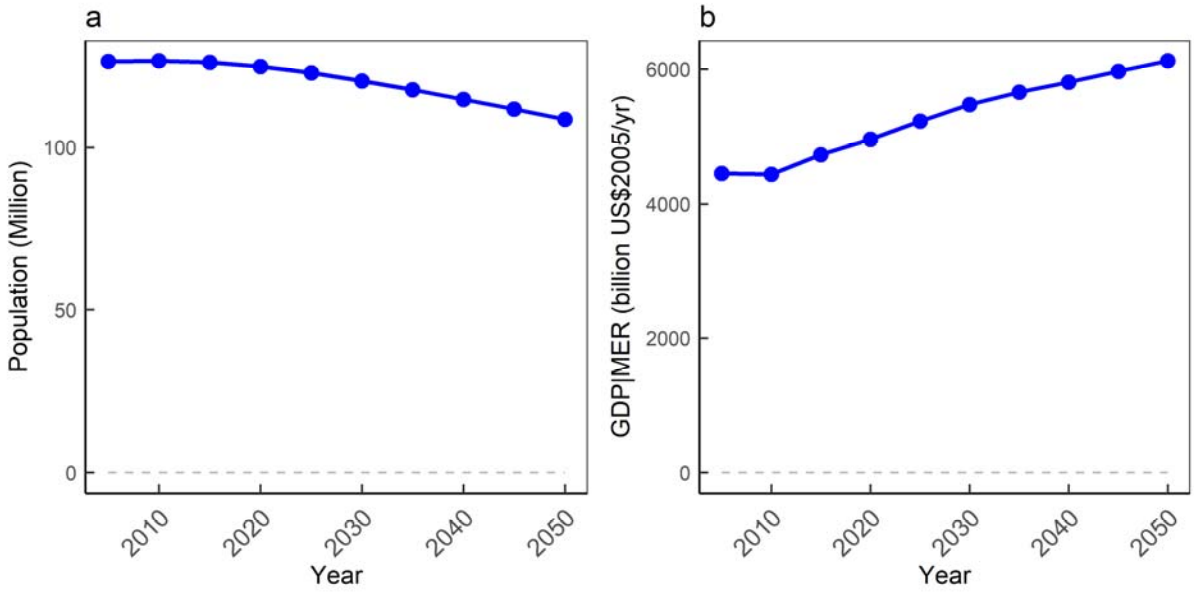

Supplementary Figure 1. Population (a) and GDP MER (Market Exchange Rates) (b) assumptions.

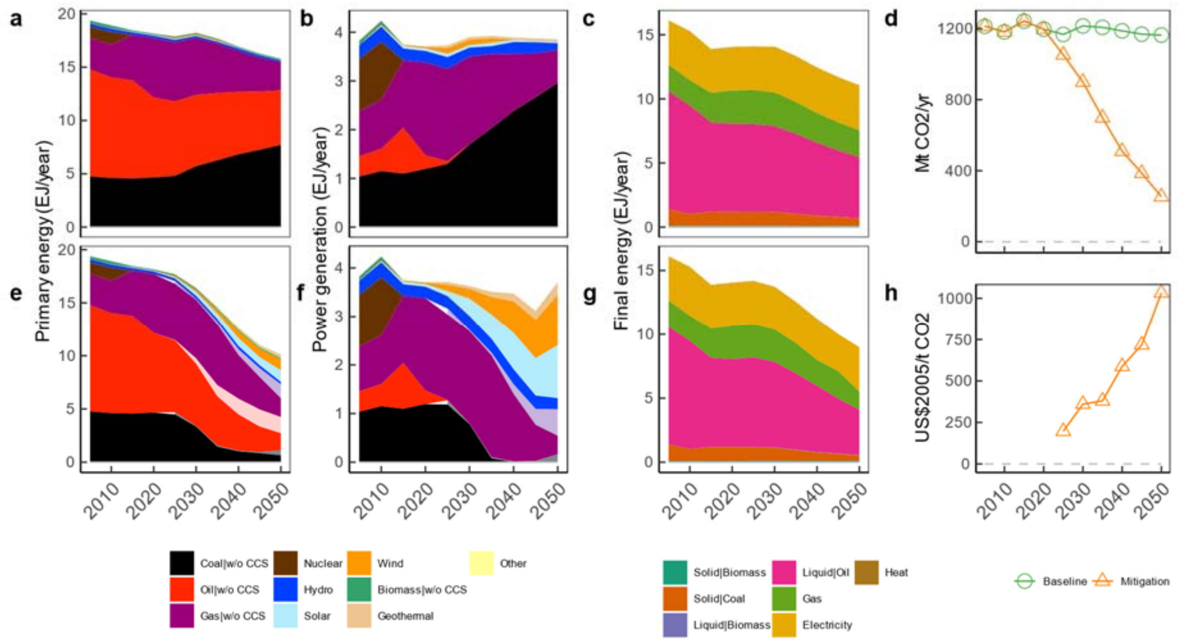

*Supplementary Figure 2. Primary energy (panels **a** and **e**), power generation (panels **b** and **f**), final energy demand (panels **c** and **g**), CO_2 emissions (panel **d**), and carbon price (panel **h**) projections for no-nuclear scenarios. Panels **a**, **b**, and **c** are baseline scenarios, whereas panels **e**, **f**, and **g** are mitigation scenarios. The other in panel **a** and **e** includes secondary energy net imports.*

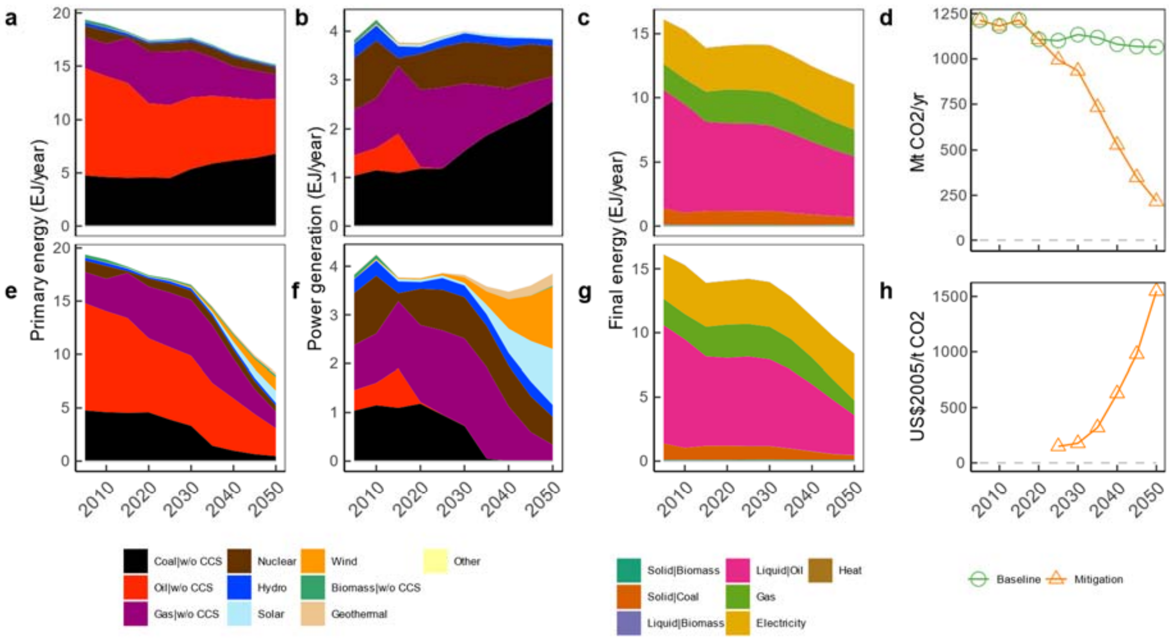

Supplementary Figure 3. *Primary energy (panels **a** and **e**), power generation (panels **b** and **f**), final energy demand (panels **c** and **g**), CO_2 emissions (panel **d**), and carbon price (panel **h**) projections for no-CCS scenarios. Panels **a**, **b**, and **c** are baseline scenarios, whereas panels **e**, **f**, and **g** are mitigation scenarios. The other in panel **a** and **e** includes secondary energy net imports.*

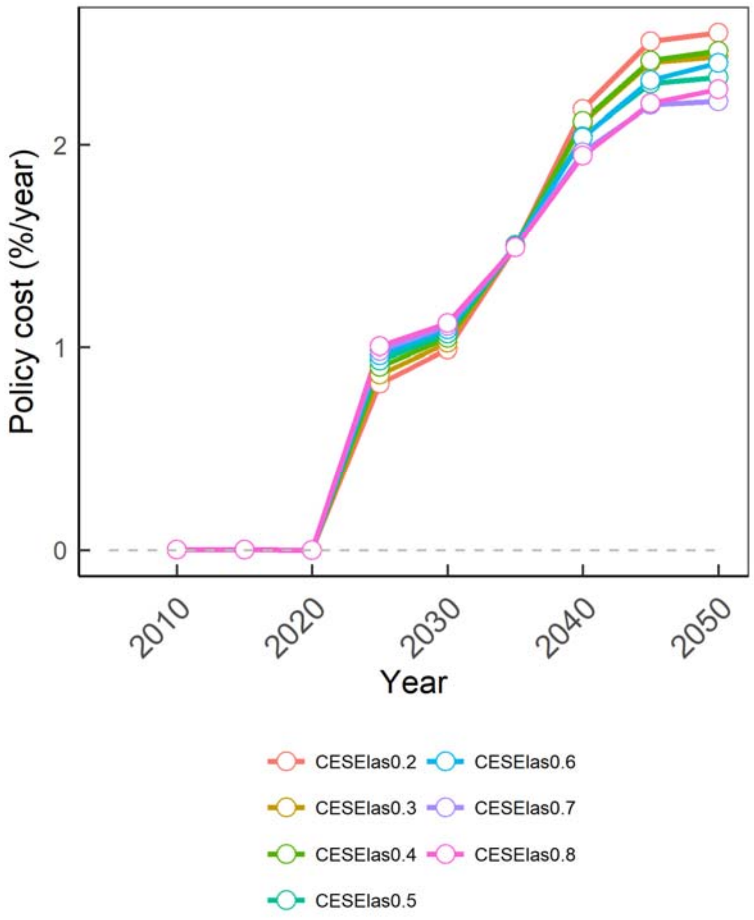

Supplementary Figure 4. The GDP loss rates associated with variation in the substitution elasticity between energy and value-added in the stand-alone model

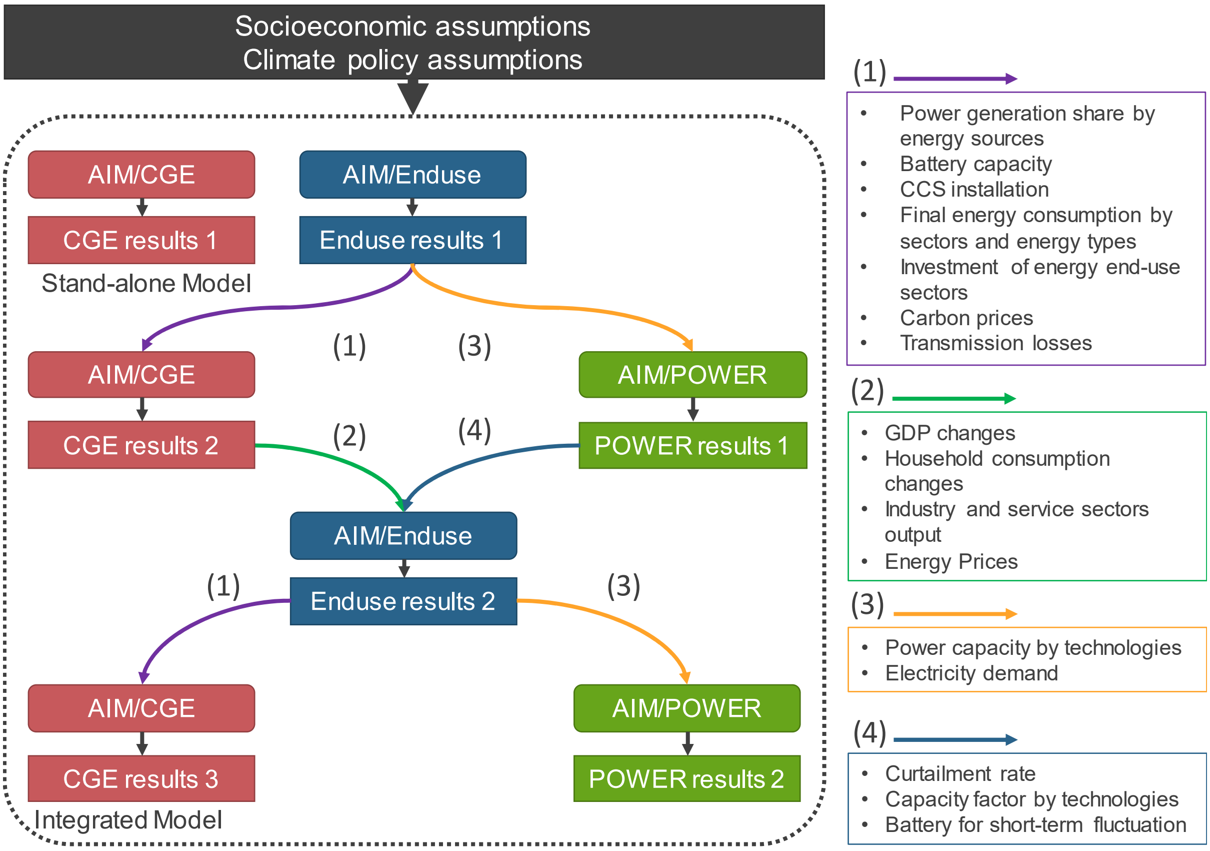

Supplementary Figure 5. Model integration strategy.

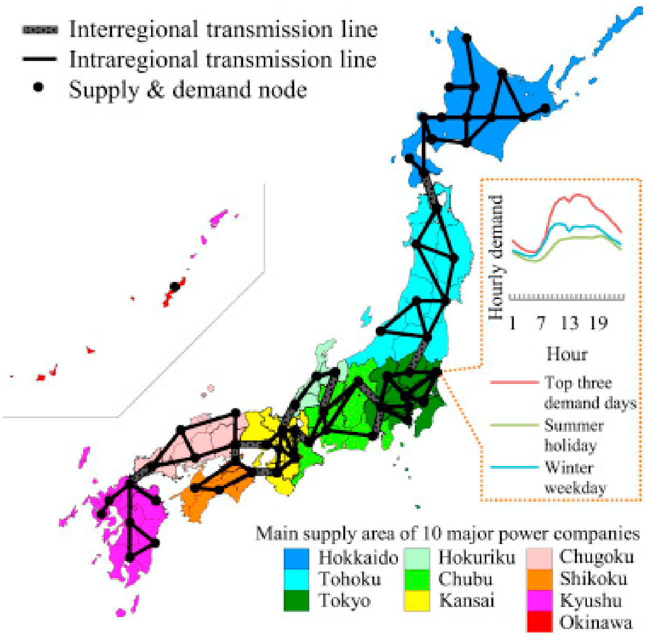

Supplementary Figure 6. Regional classification of AIM/Enduse, AIM/Power, and the power grid system (Source: Shiraki et al. 2016[1]). For AIM/Power, since a version of the model that classifies Japan into 10 regions is used in this study, intraregional transmission lines were not considered.

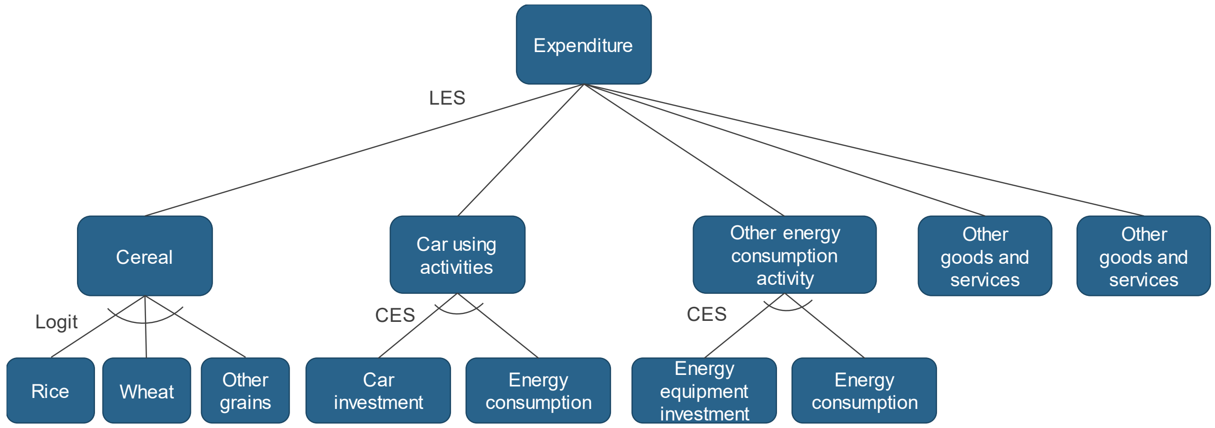

Supplementary Figure 7. Household expenditure structure.

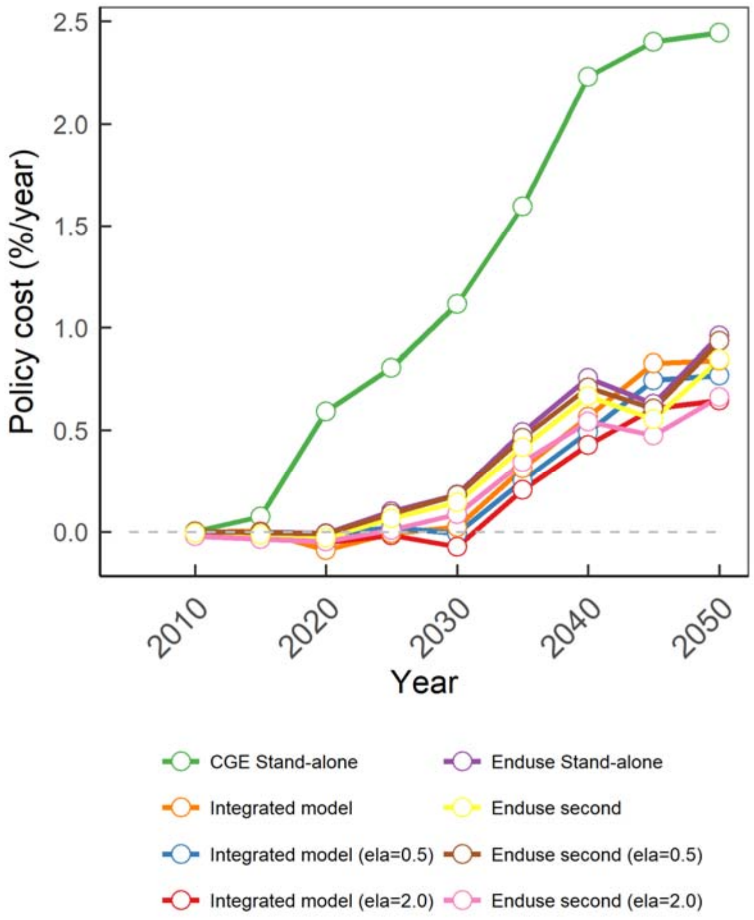

Supplementary Figure 8. Climate mitigation policy costs associated with variations in the elasticity of monetary outputs and physical energy service demand.

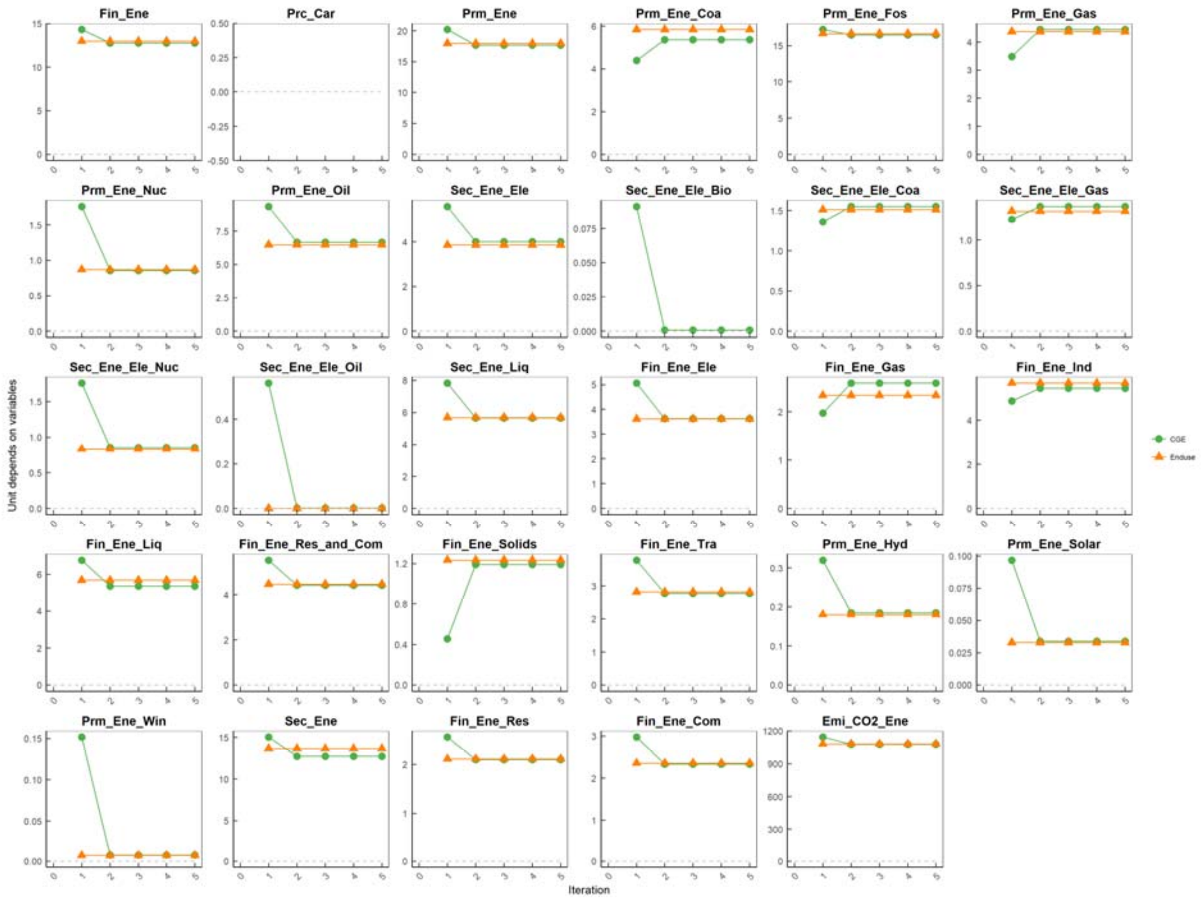

Supplementary Figure 9. Main energy, emissions and economic indicators of the baseline scenario in 2030 by iteration. Each panel illustrates an individual variable, the codes and units of which are listed in

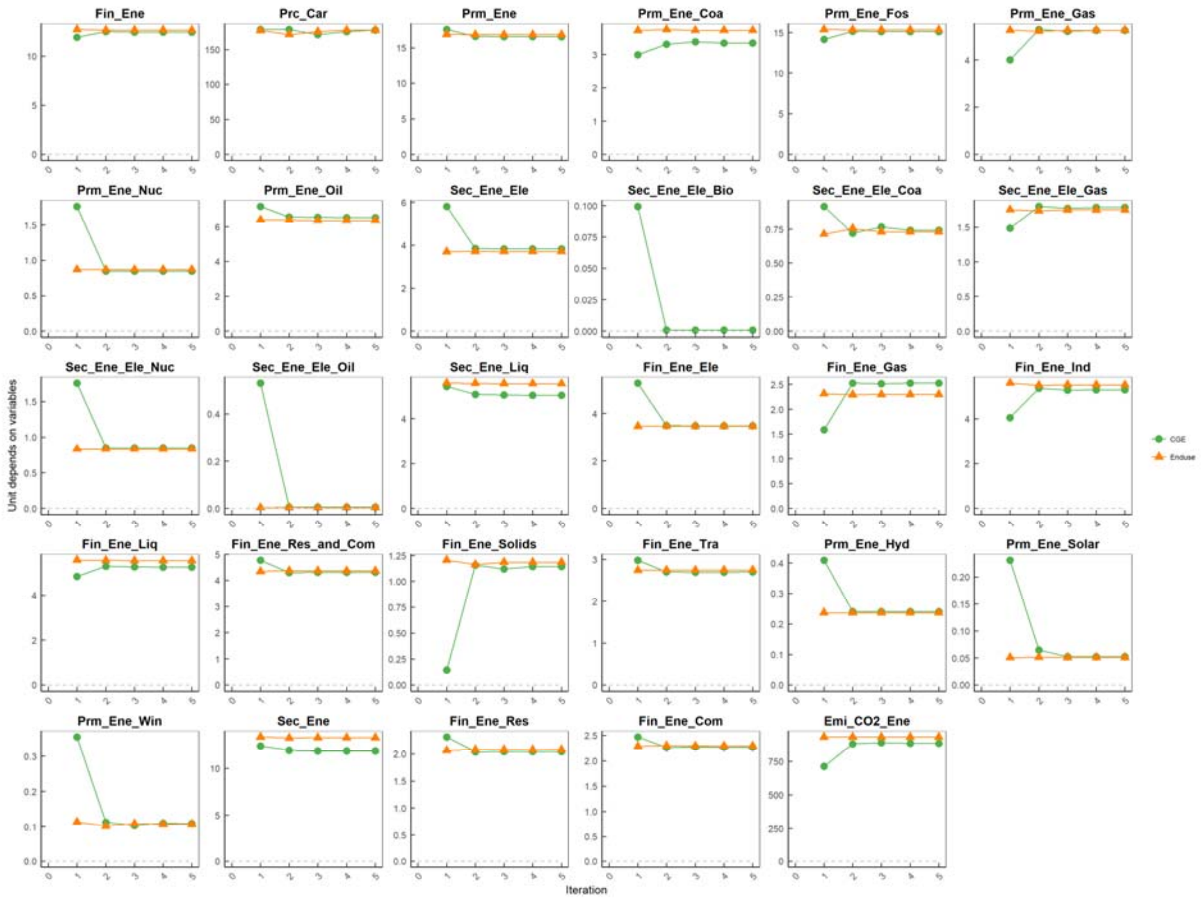

Supplementary Figure 10. Main energy, emissions and economic indicators of the mitigation scenario in 2030 by iteration. Each panel illustrates an individual variable, the codes and units of which are listed in .

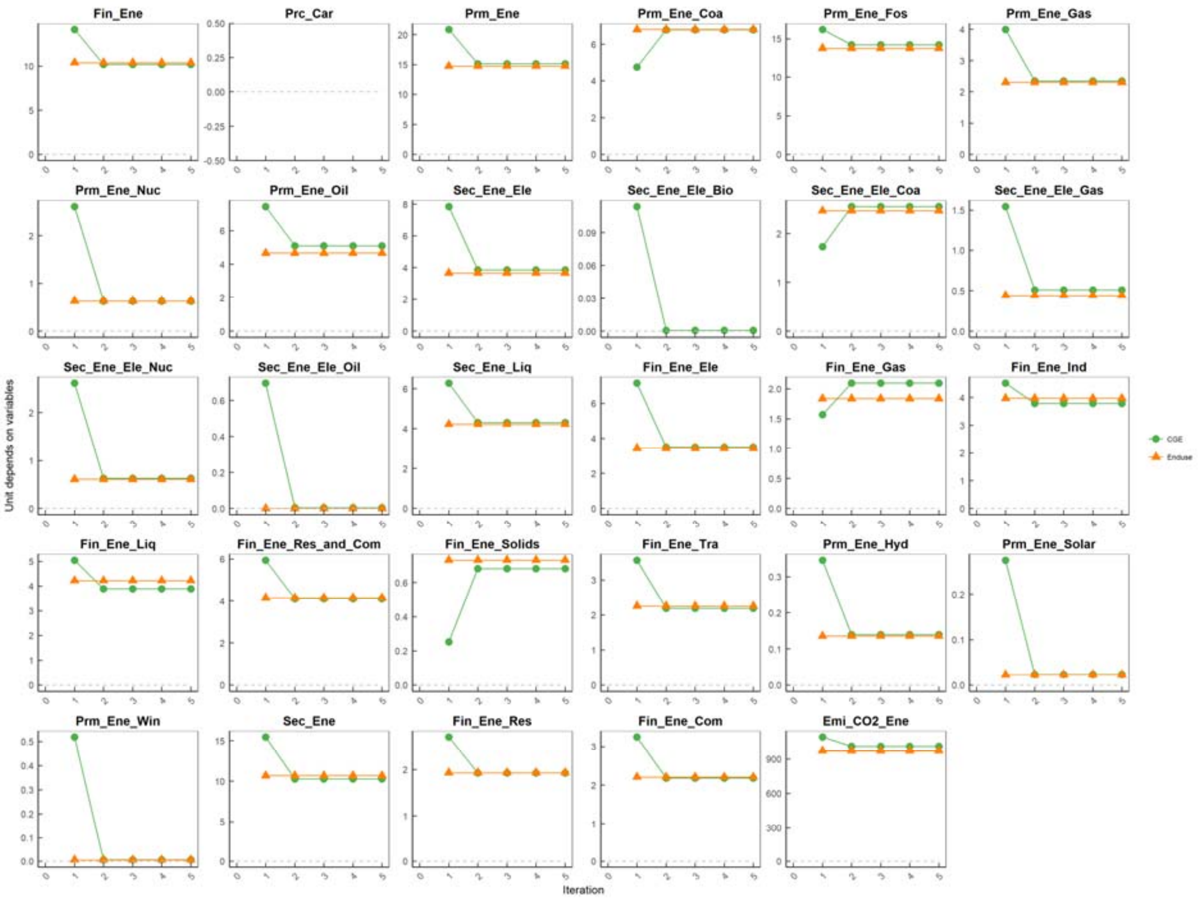

Supplementary Figure 11. Main energy, emissions and economic indicators of the baseline scenario in 2050 by iteration. Each panel illustrates an individual variable, the codes and units of which are listed in .

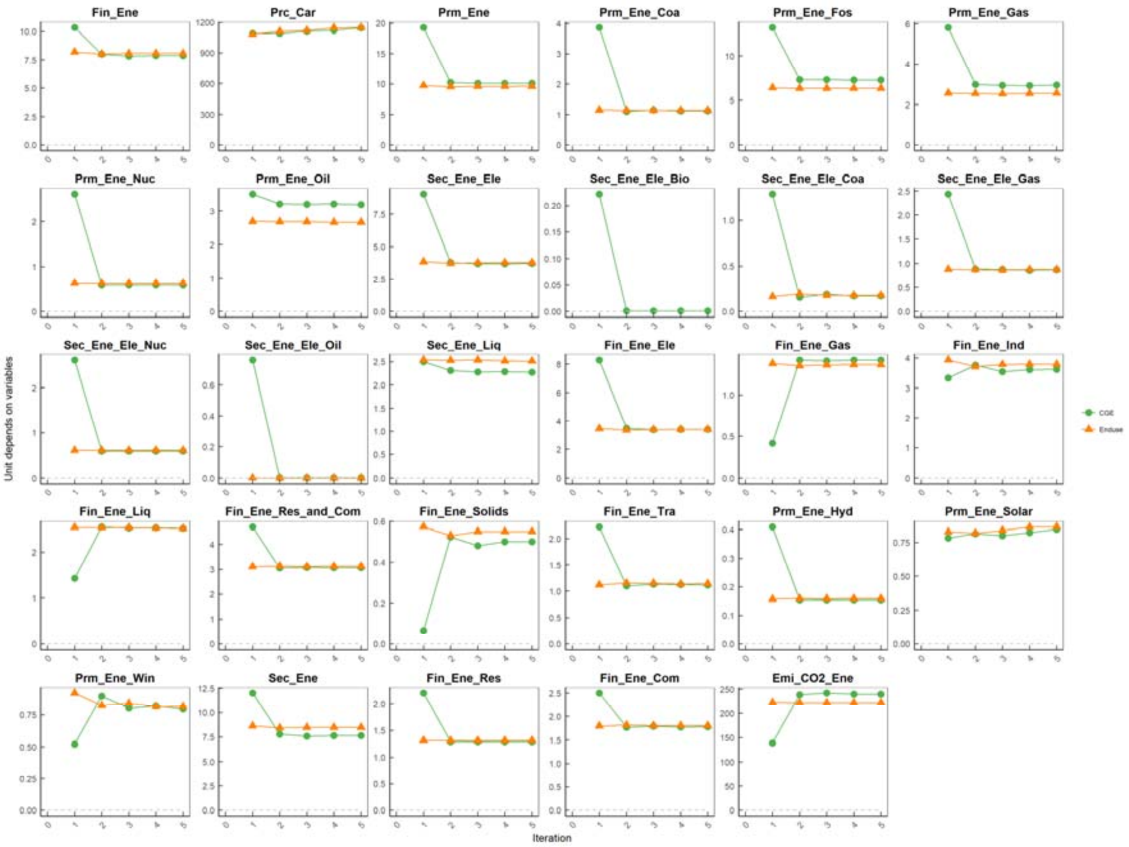

Supplementary Figure 12. Main energy, emissions and economic indicators of the mitigation scenario in 2050 by iteration. Each panel illustrates an individual variable, the codes and units of which are listed in .

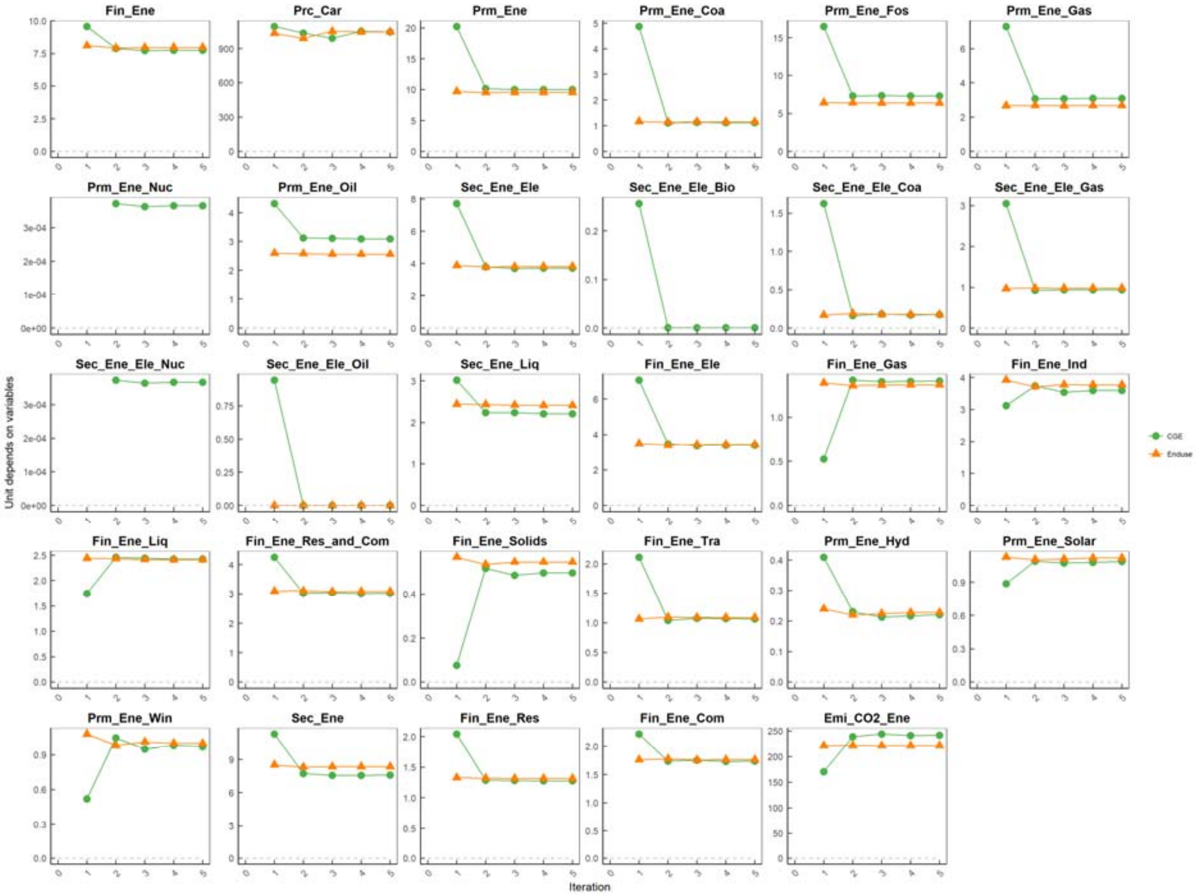

Supplementary Figure 13. Main energy, emissions and economic indicators of the mitigation scenario without nuclear in 2030 by iteration. Each panel illustrates an individual variable, the codes and units of which are listed in .

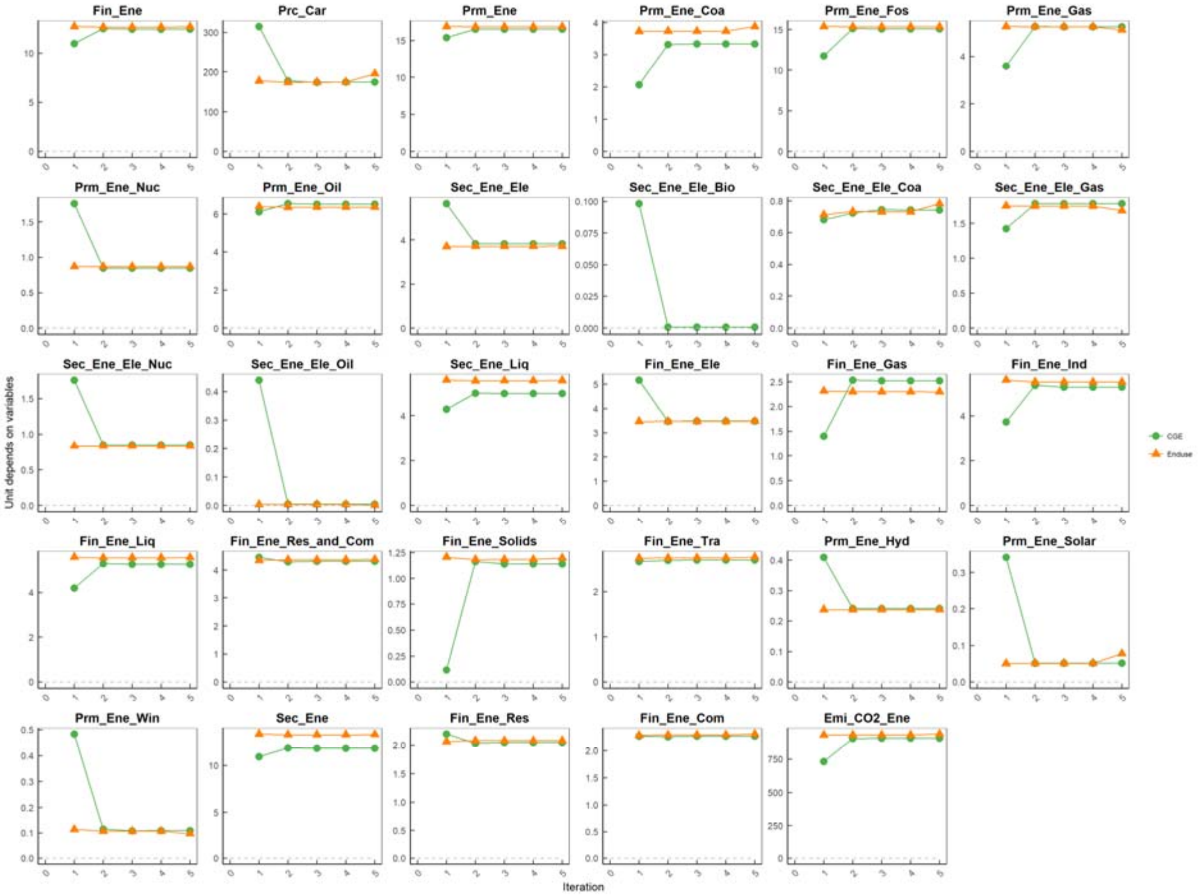

Supplementary Figure 14. Main energy, emissions and economic indicators of the mitigation scenario without CCS in 2030 by iteration. Each panel illustrates an individual variable, the codes and units of which are listed in .

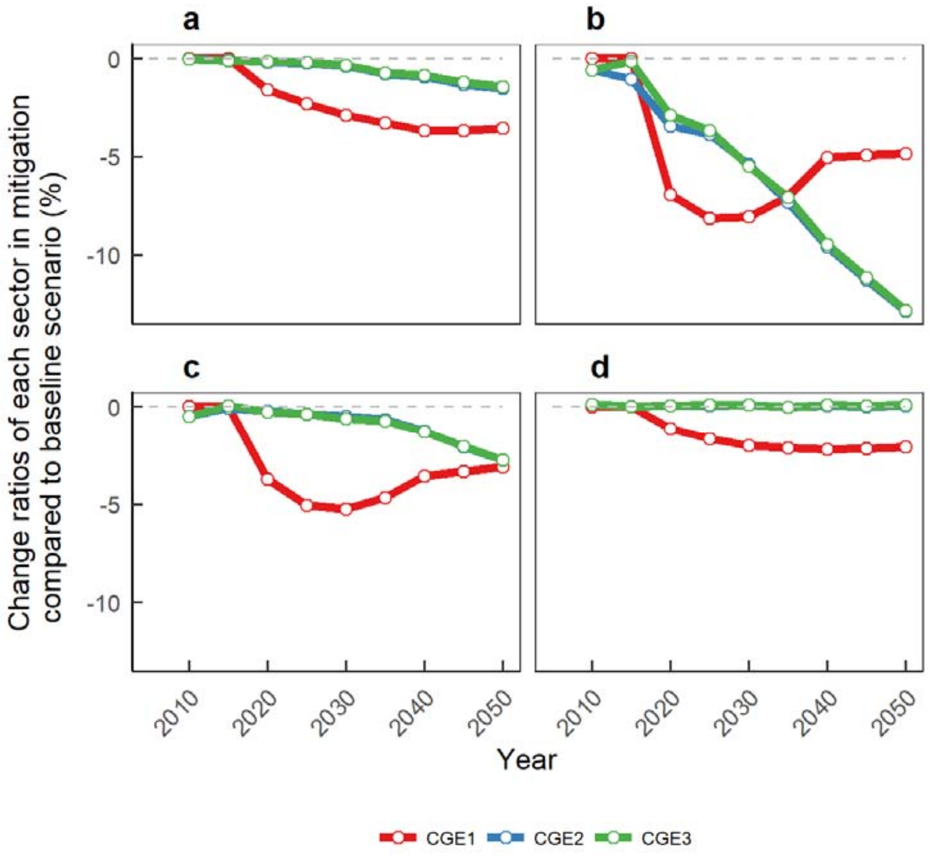

*Supplementary Figure 15. Change ratios of industrial outputs or household consumption in mitigation scenarios relative to the baseline scenario. Panels **a**, **b**, **c**, and **d** illustrate household, iron and steel, and other manufacturing and service sectors, respectively.*

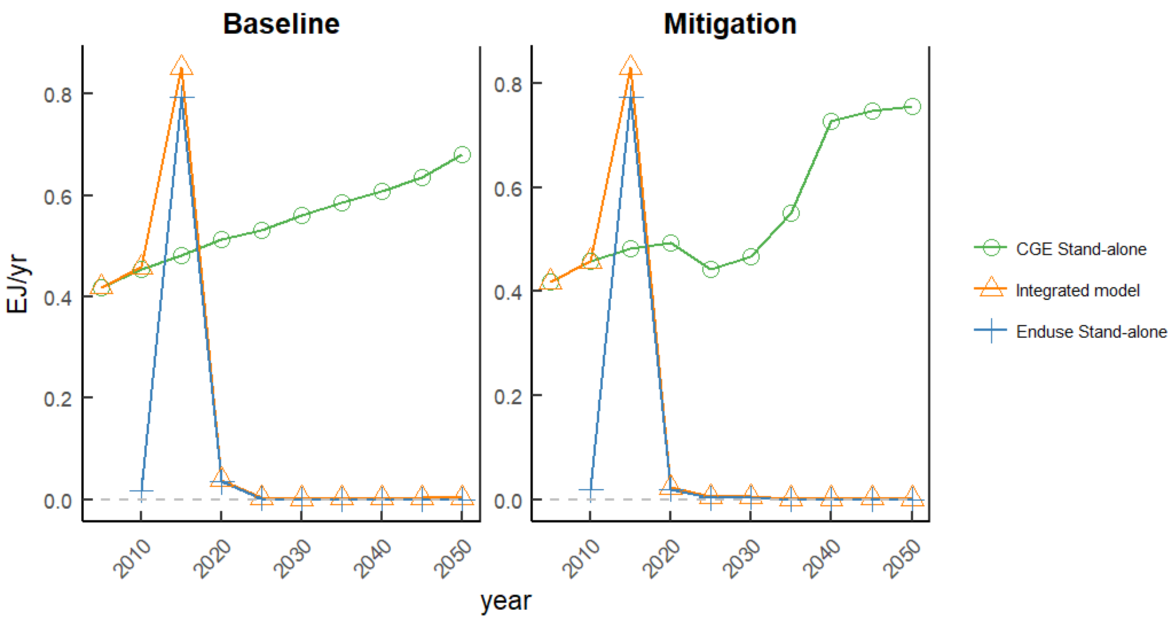

Supplementary Figure 16. Evolution of oil-fired power generation. Left and right panels show the baseline and mitigation cases.

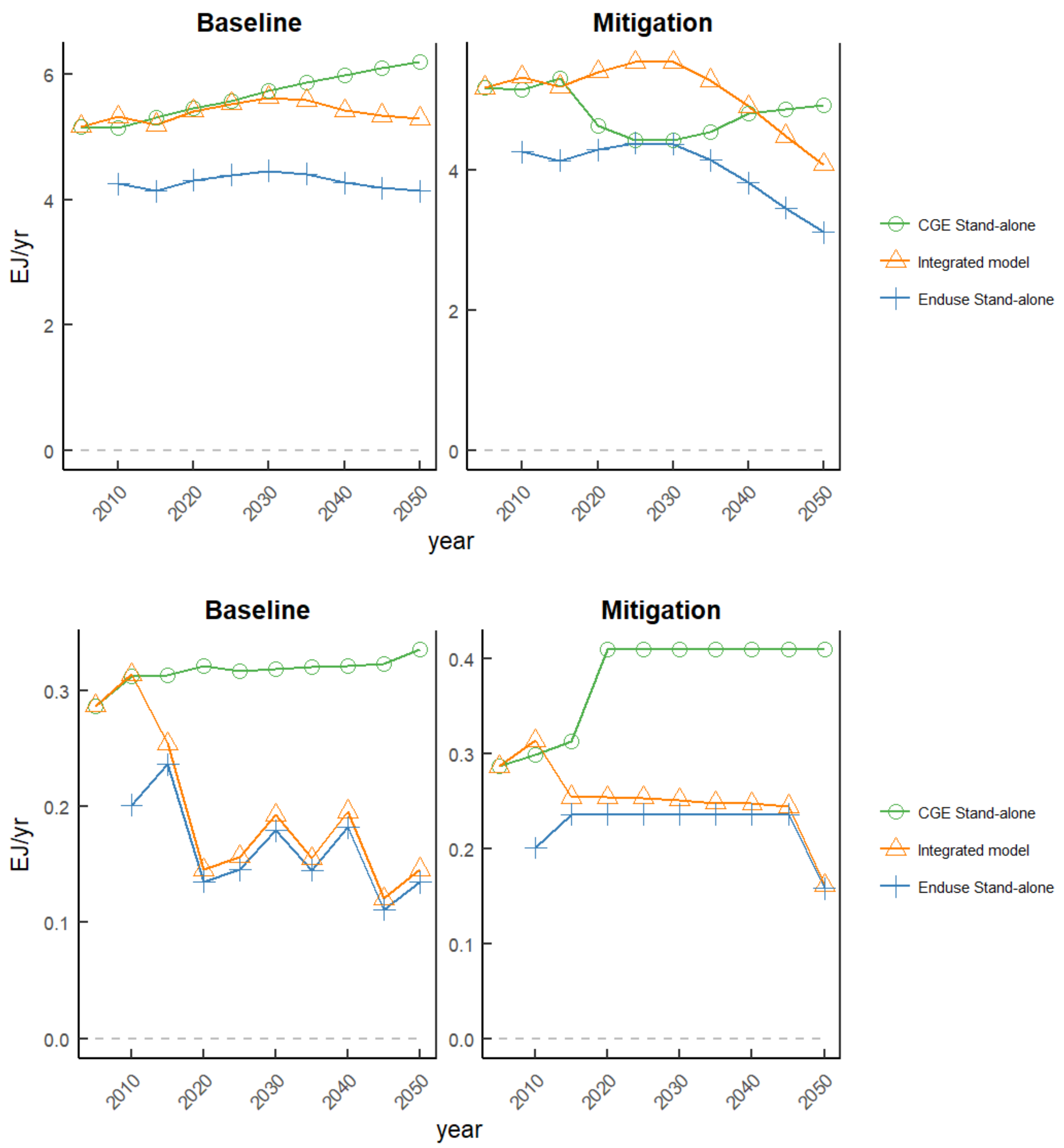

Supplementary Figure 17. Evolution of residential and commercial final energy consumption and hydropower generation. Left and right panels show the baseline and mitigation cases. Top and bottom panels show residential and commercial final energy consumption and hydropower generation, respectively.

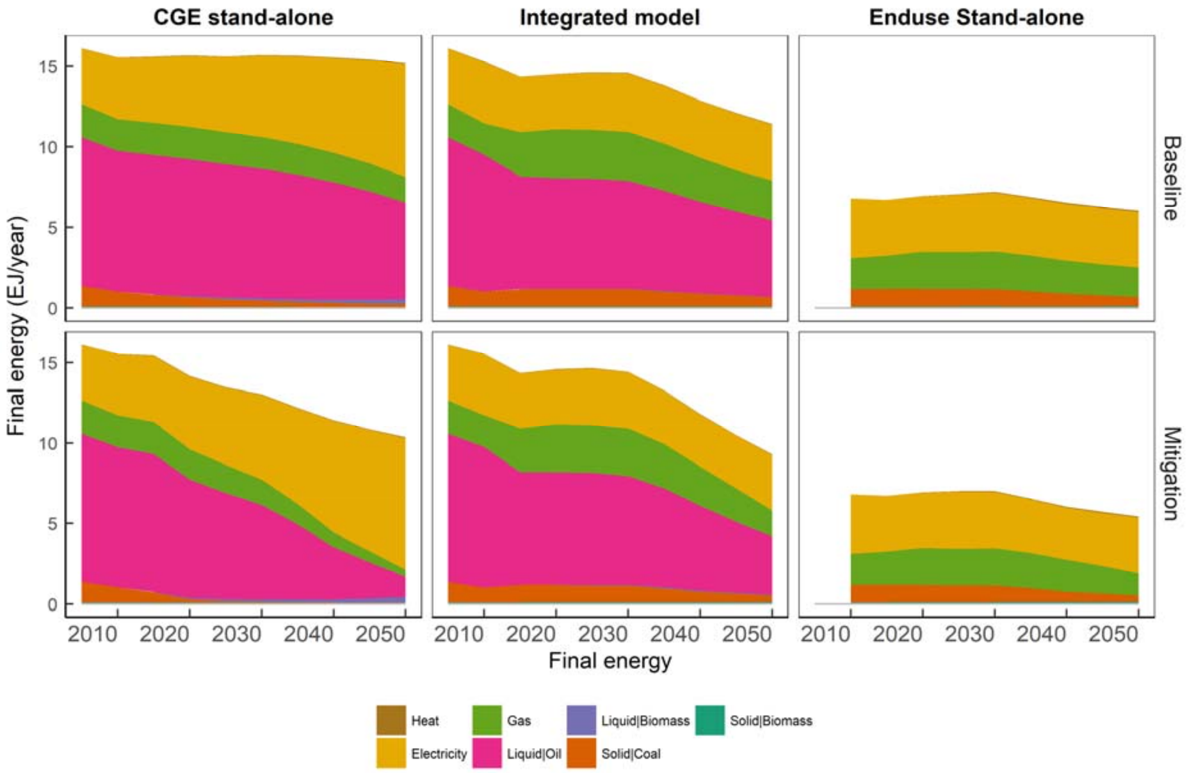

Supplementary Figure 18. Final energy consumption by energy carriers. Top and bottom panels show the baseline and mitigation cases, respectively. From left to right, CGE stand-alone, integrated model (CGE second run), and Enduse stand-alone simulation results are shown.

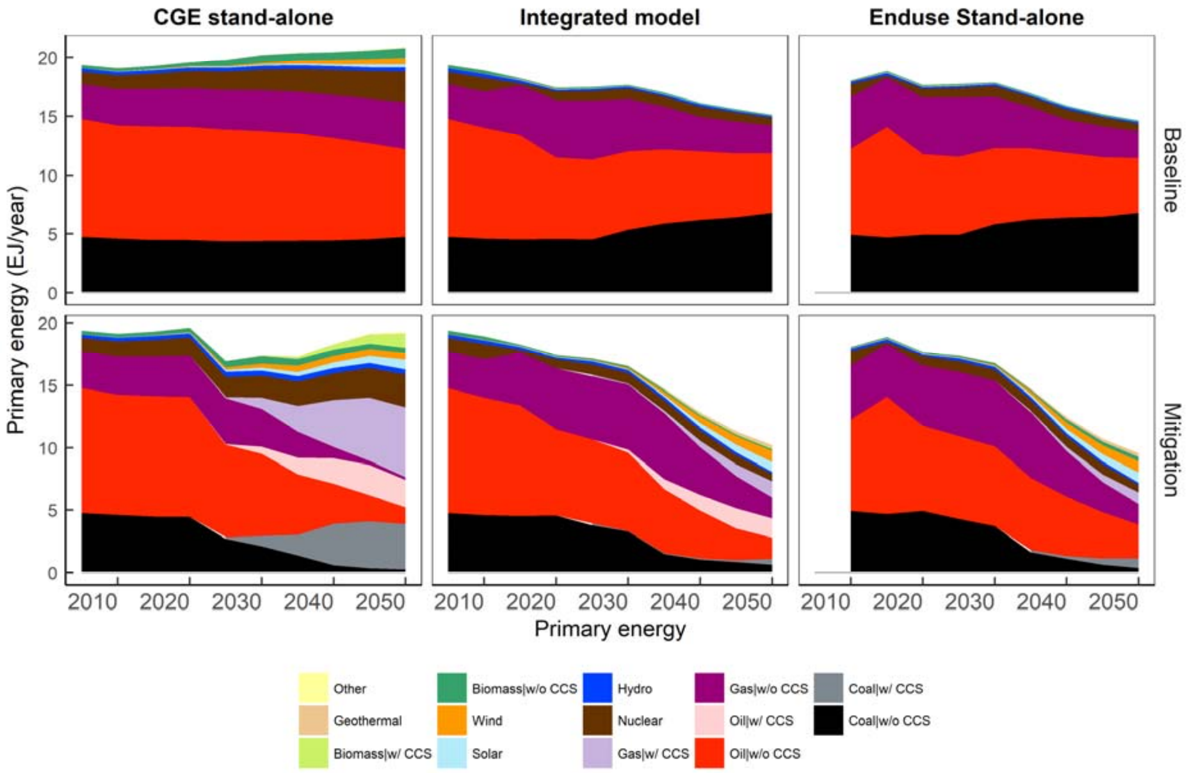

Supplementary Figure 19. Primary energy supply by energy source. Top and bottom panels show the baseline and mitigation cases, respectively. From left to right, CGE stand-alone, integrated model (CGE second run), and Enduse stand-alone simulation results are shown.

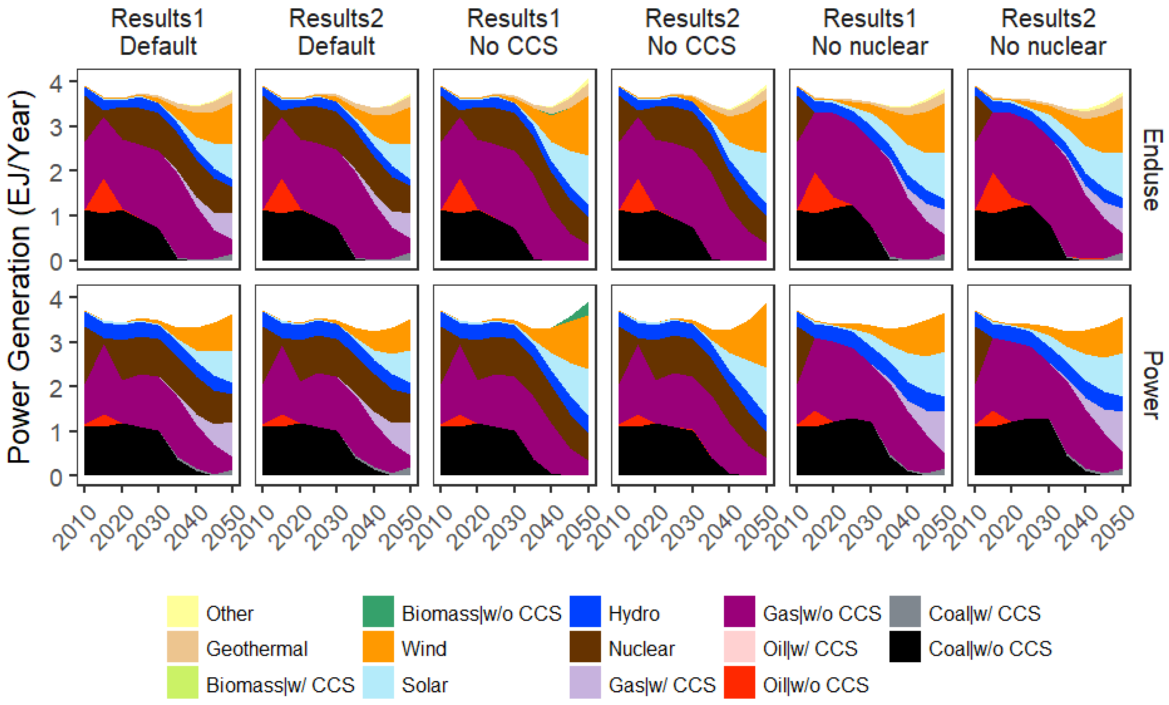

Supplementary Figure 20. Power generation by energy source generated by AIM/Enduse and AIM/Power. Top and bottom panels show AIM/Enduse and AIM/Power results, respectively. From left to right, results 1 and 2 correspond to the results of each iteration. 'Default', 'No CCS', and 'No nuclear' represent the scenario variations in technological availability.

2. Supplementary tables

Supplementary Table 1. Regression results of mitigation costs in the IPCC AR5 database[2].

	Estimate	Std. Error	t value	Pr(>|t|)	
(Intercept)	-73.791	7.594	-9.717	< 2e-16	***
550ppm	-1.238	0.217	-5.717	1.E-08	***
Year	0.037	0.004	10.076	< 2e-16	***
Asia	0.352	0.347	1.014	0.311	
Latin America	-1.527	0.356	-4.287	2.E-05	***
Middle East and Africa	0.293	0.355	0.825	0.410	
OECD	-1.368	0.346	-3.950	8.E-05	***
Reforming regions	0.546	0.383	1.425	0.154	
CGE	4.120	0.386	10.677	< 2e-16	***

1130 degrees of freedom and adjusted R-squared: 0.199

Supplementary Table 2. Literature review of CGE and energy system integration studies

Literature	Region	Integration method	Top-down model	Bottom-up model	Sector coverage of energy integration	Convergence confirmation	Electricity intermittency representation
Abrell and Rausch, 2016[3]	Europe	Soft-linking with iterative procedure	Static CGE	Electricity	Electricity	Electricity price and quantity	LDC
Andersen et al. 2019[4]	Denmark	Soft-linking with iterative procedure	Static CGE	Energy system model (TIMES)	All energy	Energy price and quantity	LDC (32 time slices per annual)
Arndt et al. 2016[5]	South Africa	Soft-linking with iterative procedure	Static CGE (SAGE)	Energy system model (TIMES)	Electricity	Electricity price and quantity	No
Boeringer et al. 2008[6]	Global	Hard-linking	Static and dynamic CGE	Electricity	Electricity	-	No
Fortes et al. 2014[7]	Portugal	Soft-linking with iterative procedure	Static CGE (GEM-E3)	Energy system model (TIMES)	All energy	Energy service demand differences	No
Helgesen et al. 2018[8]	Norway	Soft-linking with iterative procedure	Static CGE (REMES)	Energy system model (TIMES)	All energy	Commodity price and sectoral output	LDC (260 time slices per annual)
Hwang and Lee, 2015[9]	Korea	Soft-linking with iterative procedure	Static CGE	Electricity	Electricity	Electricity price and quantity	LDC (12 time slices per annual)
Krook-Riekkola et al. 2017[10]	Sweden	Soft-linking with iterative procedure	Static CGE (EMEC)	Energy system model (TIMES)	All energy	Energy consumption	LDC (12 time slices per annual)
Lanzi et al. 2012[11]	Global	Parameter calibration	Recursive dynamic CGE	World Energy Outlook	All energy	None	No
Laurent et al. 2004[12]	Switzerland	Soft-linking with iterative procedure	Static CGE	Energy system model (MARKAL)	Houshold	Carbon price	No
Sue Wing et al. 2008[13]	The US	Hard-linking	Static CGE	Electricity disaggregation	Electricity	-	No

Tapia-Ahumada et al. 2015[14]	Global	Soft-linking	Recursive dynamic CGE	Electricity	Electricity	-	Hourly
Tuladhar et al. 2009[15]	US	Soft-linking with iterative procedure	Dynamic CGE	Electricity	Electricity	Energy price and quantity	LDC (20 time slices per annual)
Vandyck et al. 2016[16]	Global	One-way Soft-linking	Recursive dynamic CGE	Energy system model (POLES)	All energy	None	No
Waisman et al. 2012[17]	Global	Recursive soft-linking	Recursive dynamic CGE	Energy system model (POLES)	All energy	None	No

Supplementary Table 3. Additional annual investment of energy end-use sector in the mitigation scenario relative to the baseline scenario computed by AIM/Enduse (unit is billion 2010US$). Negative values indicate that investment in mitigation scenario is lower than baseline scenario due to energy service demand changes and cross-sectoral effects generated by total system cost optimization.

	2010	2015	2020	2025	2030	2035	2040	2045	2050
Industry	-0.01	-0.01	-0.07	0.06	0.02	0.16	0.02	0.28	-0.26
Commercial	0.01	0	0.01	0.21	0.42	0.5	0.53	0.83	0.92
Residential	0	0	0	0.11	0.26	0.82	1.26	1.27	1.71
Transport	0	0	0.01	0.29	0.57	1.97	2.8	3.18	6.12

Supplementary Table 4. Full list of diagnostic scenarios and their GDP loss rates in 2050. Column names are sectors, and 'on' and 'off' refer to whether AIM/Enduse information is incorporated. The red and blue rows indicate the stand-alone and integrated models, respectively. Yellow and green rows indicate scenarios that include and exclude information from a single sector given by AIM/Enduse, respectively. Uncoloured rows do not appear in Table1.

	Energy Supply	Industry	Service	Transport	Residential	GDP loss rate (%)	
						2030	2050
scenario 1	off	off	off	off	off	1.1	2.4
scenario 2	off	off	off	off	on	0.9	2.3
scenario 3	off	off	off	on	off	1.1	2.4
scenario 4	off	off	off	on	on	1.0	2.3
scenario 5	off	off	on	off	off	0.6	1.7
scenario 6	off	off	on	off	on	0.5	1.5
scenario 7	off	off	on	on	off	0.7	1.6
scenario 8	off	off	on	on	on	0.6	1.5
scenario 9	off	on	off	off	off	0.4	0.8
scenario 10	off	on	off	off	on	0.3	0.6
scenario 11	off	on	off	on	off	0.5	0.7
scenario 12	off	on	off	on	on	0.4	0.6
scenario 13	off	on	on	off	off	0.1	0.4
scenario 14	off	on	on	off	on	0.0	0.3
scenario 15	off	on	on	on	off	0.2	0.3
scenario 16	off	on	on	on	on	0.1	0.2
scenario 17	On	off	off	off	off	0.9	2.2
scenario 18	on	off	off	off	on	0.9	2.3
scenario 19	on	off	off	on	off	1.1	2.4
scenario 20	on	off	off	on	on	0.9	2.5
scenario 21	on	off	on	off	off	0.5	2.0
scenario 22	on	off	on	off	on	0.4	1.8
scenario 23	on	off	on	on	off	0.7	2.3
scenario 24	on	off	on	on	on	0.5	2.2
scenario 25	on	on	off	off	off	0.4	1.2
scenario 26	on	on	off	off	on	0.3	1.0
scenario 27	on	on	off	on	off	0.6	1.3
scenario 28	on	on	off	on	on	0.4	1.2
scenario 29	on	on	on	off	off	0.0	0.8
scenario 30	on	on	on	off	on	-0.1	0.6
scenario 31	on	on	on	on	off	0.1	0.8
scenario 32	on	on	on	on	on	0.0	0.8

Supplementary Table 5. List of production sectors in AIM/CGE

Classification	Sectors	ISIC rev3 code
Energy supply (transformation) sectors	Coal mining	101, 102
	Oil mining	111, 112 (related to oil extraction), 103
	Gas mining	111, 112 (related to gas extraction)
	Petroleum refinery<br>Coal transformation	231, 232, 233
	Biomass transformation (1st generation)	-
	Biomass transformation (2nd generation with energy crop)	-
	Biomass transformation (2nd generation with residue)	-
	Gas manufacture distribution	402, 403
	Coal-fired power<br>Oil-fired power<br>Gas-fired power<br>Nuclear power<br>Hydroelectric power<br>Geothermal power<br>Photovoltaic power<br>Wind power<br>Waste biomass power<br>Other renewable energy power generation<br>Advanced biomass-power generation	401
	Hydrogen production by gas	-
	Hydrogen production by biomass	-

Supplementary Table 6 List of production sectors in AIM/CGE

Classification	Sectors	ISIC rev3 code
Agricultural sectors (Energy end-use sectors)	Rice	01
	Wheat	
	Other grains	
	Oil seed crops	
	Sugar crops	
	Other crops	
	Ruminant livestock	05
	Raw milk	
	Other livestock and fishery	
	Forestry	02
Other production sectors (Energy end-use sectors)	Mineral mining and Other quarrying	12, 13, 14
	Food products	15, 16
	Textiles and Apparel and Leather, and Wood products	17, 18, 19, 243, 20
	Paper, Paper products and Pulp	21, 2211, 2212, 2213, 2219, 222, 223
	Chemical, Plastic and Rubber products	241, 242, 25
	Mineral products nec	26
	Iron and Steel	271, 2731
	Non-Ferrous products	272, 2732
	Other Manufacturing	28, 29, 31, 33, 30, 32, 34, 35, 36, 37
	Construction	45
	Transport and communications	60, 61, 62, 63, 64
	Other service sectors	41, 50,51,52,55, 65, 66, 67, 70, 71, 72, 73, 74, 75, 80, 85, 90, 91, 92, 93, 94, 95, 99
	CCS service	-

Supplementary Table 7. List of technologies in AIM/Enduse adopted from Oshiro et al. (2015) [18]

Sector	Technology option
Industrial sector	High performance pulp washing device, High efficient black liquid boiler, Next generation coke oven, CCS for steel/cement production, DC electric furnace, Naphtha catalytic cracker, High efficient industrial boiler, Industrial heat pump, High efficient motor, High efficient agricultural device
Residential and Commercial sector	High efficient air conditioner, High efficient water heater (e.g. Heat pump water heater), Electric heat pump water heater, SOFC, High efficient lighting, High efficient appliance, High performance building envelope, Building energy management system
Transport sector	High efficient passenger vehicle, Hybrid vehicle, Plug-in hybrid electric vehicle, Battery electric vehicle (BEV), Fuel-cell electric vehicle (FCEV), CNG vehicle, Biofuel, High efficient train, High efficient ship, High efficient aircraft, Eco driving
Power generation sector	IGCC w/CCS, IGCC wo/CCS, IGFC w/CCS, IGFC wo/CCS, Advanced gas combined cycle (ACC) w/CCS, ACC wo/CCS, Fuel cell gas combined cycle w/ or wo/CCS, Nuclear, Onshore wind power, Offshore wind power, Solar PV, Geothermal, Bioenergy, Hydropower, Pumped hydro, Reinforcing electricity interconnection capacity, Hydrogen generation by electrolysis
Other sectors	Reduced fertilization, HFCs leakage reduction and recovery, Reduced municipal solid waste

Supplementary Table 8. List of technoeconomic information of power sector in AIM./Enduse.

Variable	Unit	2010	2015	2020	2025	2030	2035	2040	2045	2050
Capital Cost|Biomass|w/ CCS	US$2010/kW	7540	8063	8063	8063	8063	8063	8063	8063	8063
Capital Cost|Biomass|w/o CCS	US$2010/kW	3813	4336	4336	4336	4336	4336	4336	4336	4336
Capital Cost|Coal|w/ CCS	US$2010/kW	4338	4338	4338	4338	4338	4338	4338	4338	4338
Capital Cost|Coal|w/o CCS	US$2010/kW	2704	2704	2704	2704	2704	2704	2704	2704	2704
Capital Cost|Gas|w/ CCS	US$2010/kW	2174	2174	2174	2174	2174	2174	2174	2174	2174
Capital Cost|Gas|w/o CCS	US$2010/kW	1122	1122	1122	1122	1122	1122	1122	1122	1122
Capital Cost|Geothermal	US$2010/kW	8716	8607	8607	8607	8607	8607	8607	8607	8607
Capital Cost|Hydro	US$2010/kW	8716	8716	8716	8716	8716	8716	8716	8716	8716
Capital Cost|Nuclear	US$2010/kW	4506	4721	4721	4721	4721	4721	4721	4721	4721
Capital Cost|Solar|PV	US$2010/kW	5704	4031	3035	2588	2141	2065	1990	1914	1838
Capital Cost|Wind|Offshore	US$2010/kW	5443	5704	5449	5194	4940	4797	4655	4513	4370
Capital Cost|Wind|Onshore	US$2010/kW	3046	3145	2918	2725	2531	2531	2531	2490	2449
Lifetime|Biomass|w/ CCS	years	40	40	40	40	40	40	40	40	40
Lifetime|Biomass|w/o CCS	years	40	40	40	40	40	40	40	40	40
Lifetime|Coal|w/ CCS	years	40	40	40	40	40	40	40	40	40
Lifetime|Coal|w/o CCS	years	40	40	40	40	40	40	40	40	40
Lifetime|Gas|w/ CCS	years	40	40	40	40	40	40	40	40	40
Lifetime|Gas|w/o CCS	years	40	40	40	40	40	40	40	40	40
Lifetime|Geothermal	years	40	40	40	40	40	40	40	40	40
Lifetime|Hydro	years	80	80	80	80	80	80	80	80	80
Lifetime|Nuclear	years	40	40	40	40	40	40	40	40	40
Lifetime|Solar|PV	years	15	15	15	15	15	15	15	15	15
Lifetime|Wind|Offshore	years	15	15	15	15	15	15	15	15	15
Lifetime|Wind|Onshore	years	15	15	15	15	15	15	15	15	15
OM Cost|Fixed|Biomass|w/ CCS	US$2010/kW/yr	982	982	982	982	982	982	982	982	982
OM Cost|Fixed|Biomass|w/o CCS	US$2010/kW/yr	290	290	290	290	290	290	290	290	290
OM Cost|Fixed|Coal|w/ CCS	US$2010/kW/yr	303	303	303	303	303	303	303	303	303
OM Cost|Fixed|Coal|w/o CCS	US$2010/kW/yr	106	106	106	106	106	106	106	106	106
OM Cost|Fixed|Gas|w/ CCS	US$2010/kW/yr	159	159	159	159	159	159	159	159	159
OM Cost|Fixed|Gas|w/o CCS	US$2010/kW/yr	34	34	34	34	34	34	34	34	34
OM Cost|Fixed|Geothermal	US$2010/kW/yr	354	354	354	354	354	354	354	354	354
OM Cost|Fixed|Hydro	US$2010/kW/yr	379	379	379	379	379	379	379	379	379
OM Cost|Fixed|Nuclear	US$2010/kW/yr	200	200	200	200	200	200	200	200	200
OM Cost|Fixed|Solar|PV	US$2010/kW/yr	39	39	39	39	39	39	39	39	39
OM Cost|Fixed|Wind|Offshore	US$2010/kW/yr	241	241	241	241	241	241	241	241	241
OM Cost|Fixed|Wind|Onshore	US$2010/kW/yr	64	64	64	64	64	64	64	64	64

Supplementary Table 9. Assumed power plant capacity by construction year (Unit is GW)

Construction year	-1970	1971 -75	1976 -80	1981 -85	1986 -90	1991 -95	1996 -00	2001 -05	2006 -10
Coal|w/o CCS	0.2	4.4	2.5	4.1	4.1	8.3	10.1	10.7	2.8
Gas|w/o CCS	0.6	14.5	9.0	6.7	7.9	4.5	12.9	4.3	9.6
Oil|w/o CCS	12.8	15.0	7.8	8.1	3.3	2.3	3.4	2.3	1.5
Hydro	16.1	1.0	1.0	1.1	0.6	0.4	0.2	0.2	0.1
Nuclear		12.1	5.7	3.4	6.5	11.1	4.7	2.2	3.2
Biomass|w/o CCS									0.2
Geothermal	0.0	0.0	0.0	0.1	0.1	0.2	0.1		0.0
Solar|PV						0.0	0.2	0.9	1.9
Wind|Onshore					0.0	0.0	0.1	0.9	1.4

Supplementary Table 10. Assumptions on fuel prices

Variable	Unit	2010	2020	2030	2040	2050
Price|Coal	US$2010/GJ	4.1	3.9	3.8	3.4	3.3
Price|Gas	US$2010/GJ	9.9	9.3	11.2	11.3	11.4
Price|Oil	US$2010/GJ	12.8	14.9	17.3	15.9	14.7

Supplementary Table 11. Mapping of household consumption goods and general goods categories

Household consumption goods category	General goods category
Car related consumption	Petroleum products<br>Electricity<br>Biofuel<br>Other Manufacturing
Non-car related energy	Coal<br>Oil<br>Gas<br>Petroleum products<br>Coal products<br>Biofuel<br>Town gas<br>Electricity<br>Hydrogen<br>Other Manufacturing
Cereal	Rice<br>Wheat<br>Other grains
Oil seed crops	Oil seed crops
Sugar crops	Sugar crops
Other crops	Other crops
Ruminant livestock	Ruminant livestock
Raw milk	Raw milk
Other livestock and fishery	Other livestock and fishery
Forestry	Forestry
Mineral mining and Other quarrying	Mineral mining and Other quarrying
Food products	Food products
Textiles and Apparel and Leather	Textiles and Apparel and Leather
Wood products	Wood products
Paper, Paper products and Pulp	Paper, Paper products and Pulp
Chemical, Plastic and Rubber products	Chemical, Plastic and Rubber products
Iron and Steel	Iron and Steel
Non-Ferrous products	Non-Ferrous products
Construction	Construction
Transport and communications	Transport and communications
Other services	Other services

Supplementary Table 12. Error rates (%) between AIM/CGE and AIM/Enduse models. CGE1, CGE2, CGE3, End1, and End2 correspond to the results shown in Supplementary Figure 5 results for each model output. For example, CGE_results1 and Enduse results1 are CGE1 and End1 respectively.

	Baseline			Mitigation		
	End1_CGE1	End1_CGE2	End2_CGE3	End1_CGE1	End1_CGE2	End2_CGE3
Final Energy (EJ/yr)	7%	1%	1%	4%	1%	1%
Primary Energy (EJ/yr)	8%	1%	1%	13%	1%	1%
Primary Energy|Coal (EJ/yr)	11%	2%	2%	26%	6%	4%
Primary Energy|Fossil Fuel (EJ/yr)	4%	1%	1%	11%	2%	2%
Primary Energy|Gas (EJ/yr)	13%	1%	1%	15%	2%	2%
Primary Energy|Nuclear (EJ/yr)	34%	1%	1%	34%	1%	1%
Primary Energy|Oil (EJ/yr)	15%	2%	2%	6%	2%	2%
Secondary Energy|Electricity (EJ/yr)	20%	2%	2%	25%	1%	1%
Secondary Energy|Electricity|Coal (EJ/yr)	11%	1%	1%	43%	5%	1%
Secondary Energy|Electricity|Gas (EJ/yr)	21%	2%	2%	18%	1%	1%
Secondary Energy|Electricity|Non-Biomass Renewables (EJ/yr)	0%	0%	0%	0%	0%	0%
Secondary Energy|Electricity|Nuclear (EJ/yr)	35%	1%	1%	35%	1%	1%
Secondary Energy|Electricity|Oil (EJ/yr)	75%	15%	15%	75%	37%	11%
Secondary Energy|Liquids (EJ/yr)	13%	0%	0%	3%	4%	4%
Final Energy|Electricity (EJ/yr)	19%	0%	0%	25%	0%	0%
Final Energy|Gases (EJ/yr)	5%	4%	4%	20%	3%	4%
Final Energy|Industry (EJ/yr)	4%	2%	2%	10%	2%	2%
Final Energy|Liquids (EJ/yr)	7%	2%	2%	9%	2%	2%
Final Energy|Residential and Commercial (EJ/yr)	10%	0%	0%	8%	1%	1%
Final Energy|Solids (EJ/yr)	32%	2%	2%	58%	2%	2%
Final Energy|Transportation (EJ/yr)	13%	1%	1%	9%	1%	1%
Primary Energy|Hydro (EJ/yr)	29%	1%	1%	25%	7%	1%
Primary Energy|Solar (EJ/yr)	57%	1%	1%	13%	1%	1%
Primary Energy|Wind (EJ/yr)	86%	1%	1%	22%	1%	1%
Secondary Energy (EJ/yr)	8%	2%	2%	6%	4%	4%
Final Energy|Residential (EJ/yr)	9%	0%	0%	10%	1%	1%
Final Energy|Commercial (EJ/yr)	11%	0%	0%	7%	0%	0%
Emissions|CO2|Energy and Industrial Processes (Mt CO2/yr)	4%	1%	1%	10%	2%	1%

Supplementary Table 13. Variable code and unit list

Code	Variable	Unit
Fin_Ene	Final Energy	EJ/yr
Prc_Car	Price|Carbon	US$2005/t CO2
Prm_Ene	Primary Energy	EJ/yr
Prm_Ene_Coa	Primary Energy|Coal	EJ/yr
Prm_Ene_Fos	Primary Energy|Fossil Fuel	EJ/yr
Prm_Ene_Gas	Primary Energy|Gas	EJ/yr
Prm_Ene_Nuc	Primary Energy|Nuclear	EJ/yr
Prm_Ene_Oil	Primary Energy|Oil	EJ/yr
Sec_Ene_Ele	Secondary Energy|Electricity	EJ/yr
Sec_Ene_Ele_Bio	Secondary Energy|Electricity|Biomass	EJ/yr
Sec_Ene_Ele_Coa	Secondary Energy|Electricity|Coal	EJ/yr
Sec_Ene_Ele_Gas	Secondary Energy|Electricity|Gas	EJ/yr
Sec_Ene_Ele_Nuc	Secondary Energy|Electricity|Nuclear	EJ/yr
Sec_Ene_Ele_Oil	Secondary Energy|Electricity|Oil	EJ/yr
Sec_Ene_Liq	Secondary Energy|Liquids	EJ/yr
Fin_Ene_Ele	Final Energy|Electricity	EJ/yr
Fin_Ene_Gas	Final Energy|Gases	EJ/yr
Fin_Ene_Ind	Final Energy|Industry	EJ/yr
Fin_Ene_Liq	Final Energy|Liquids	EJ/yr
Fin_Ene_Res_and_Com	Final Energy|Residential and Commercial	EJ/yr
Fin_Ene_Solids	Final Energy|Solids	EJ/yr
Fin_Ene_Tra	Final Energy|Transportation	EJ/yr
Prm_Ene_Hyd	Primary Energy|Hydro	EJ/yr
Prm_Ene_Solar	Primary Energy|Solar	EJ/yr
Prm_Ene_Win	Primary Energy|Wind	EJ/yr
Sec_Ene	Secondary Energy	EJ/yr
Fin_Ene_Res	Final Energy|Residential	EJ/yr
Fin_Ene_Com	Final Energy|Commercial	EJ/yr
Emi_CO2_Ene	Emissions|CO2|Energy	Mt CO2/yr

3. Supplementary notes

Supplementary Note 1. Climate mitigation costs in the IPCC AR5 database

We investigated the IPCC AR5 database to determine whether climate mitigation costs in multi-sector CGE models are higher than those in other types of models. The AR5 database includes various scenarios that cannot be dealt with equally. For example, the scenarios developed under EMF27 (Energy Modeling Forum) include assumptions about technological variations. Climate targets also differ among scenarios. Therefore, we first selected scenarios that do not have any restrictions on technological availability from EMF27. Then, the climate targets were classified into two categories equivalent to 450 and 550 ppm CO_2 at the end of this century. We then applied simple regression analysis, as follows:

$$MCR_s = \sum_{(s,l)\in SL} b_l Y_l + e_s$$

where MCR_s is the climate mitigation cost (%) for scenario s; b_l is a dummy parameter representing a set of model classifications (multi-sector CGE or not), years, regions (five regions, Asia, Latin America, Middle East-Africa, OECD, and Reforming region + global), and climate targets (450 or 550ppm); Y_l is an estimated variable; and e_s is an error term. Here, we take climate mitigation cost to represent GDP losses as a standard metric, but for models that do not include GDP losses, additional energy system costs or area under MAC (marginal abatement cost) are used. The regression results show that the CGE model is a positive factor with a statistically significant *t*-value.

Supplementary Note 2. Mathematical formula of CGE model

In this section, we describe the mathematical formulas in the AIM/CGE model, which is particularly relevant to energy consumption and power generation. We use different production functions between energy end-use and power generation sectors and they are described as below.

1) Energy end-use sectors other than household sector in CGE model

We begin with the representation of the energy end-use sectors (Supplementary Table 5). As many of other CGE models, AIM/CGE assumes KL-E type multi-nested CES production function[19]. The value-added and energy composite inputs are determined by multiplying a coefficient by the output from the energy end-use sectors (Equation 1) that is calibrated. Then, value-added and energy composite are combined with the CES function (Equation 2). Labour and capital inputs are further nested in the CES function, as shown in Equation 3.

$$QVAE_a^B = ivae_a \cdot QA_a^B \quad (1)$$

$$QVAE_a^B = \alpha ae_a \left(\beta ae_a \cdot QVA_a^{B^{-\rho ae_a}} + (1-\beta ae_a) \cdot (at_a \cdot QENE_a^B)^{-\rho ae_a}\right)^{\frac{1}{-\rho ae_a}} \quad (2)$$

$$QVA_a^B = tfp \cdot \alpha f_a \left(\beta f_a \cdot QF_{"capital",a}^{B\ \ -\rho ae_a} + (1-\beta f_a) \cdot QF_{"labor",a}^{B\ \ -\rho f_a}\right)^{\frac{1}{-\rho f_a}} \quad (3)$$

where
$a \in A$ is a set of production activities,
QA_a^B is an output of sector a in the baseline scenarios (monetary unit),
$QVAE_a^B$ is the composite of value-added and energy of sector a in the baseline scenarios (monetary unit),
QVA_a^B is value-added of sector a in baseline scenarios (monetary unit),
$QENE_a^B$ is the total energy inputs including any energy carriers (e.g. gas, liquids, and electricity) of sector a in baseline scenarios (physical unit),
$QF_{f,a}^B$ is the primary factor inputs of factor f and sector a in baseline scenarios (f = capital or labor) (monetary unit),
$ivae_a$ is an input coefficient of the output of sector a,
αae_a is the scale parameter of the CES function for value-added and energy aggregates,
βae_a is the share parameter of the CES function for value-added and energy aggregates,
ρae_a is the exponent parameter of the CES function for value-added and energy aggregates,
αf_a is the scale parameter of the CES function for primary factor aggregates,
βf_a is the share parameter of the CES function for primary factor aggregates,

ρf_a is the exponent parameter of the CES function for primary factor aggregates,
at_a is an autonomous energy efficiency improvement parameter, and
tfp is a parameter that represents the economy wide total factor productivity that is calibrated in the baseline scenarios by hitting the target GDP and the calibrated values are adopted in the mitigation scenarios.

The energy carriers shares in the energy end-use sectors are calculated by using McFadden's (1981)[20] logit share equation. The concept underlying the logit is that the decision makers determine the share of each element under a certain probability distribution function. This function form is applied in some other IAMs (GCAM[21] and IMAGE[22]). For further theoretical discussion of the logit sharing mechanism, see Clarke and Edmonds (1993)[23]. $QENE$ shown above is the sum of the individual energy carriers determined by this equation.

$$SHENE_{c,a} = \frac{ae_c \cdot \delta_{c,a}^{en} \cdot PQ_{c,a}^{-\beta^{el}}}{\sum_{cp \in CENE} ae_{cp} \cdot \delta_{cp,a}^{en} \cdot PQ_{cp,a}^{-\beta^{el}}} \quad c \in CENE, a \in AEnd \tag{5}$$

Where
$a \in AEnd$ is energy end-use production activities and a subset of the production activities (e.g. industry, transport and so on),
$c \in CENE$ is set of energy commodities (coal, petroleum products and so on), which is a subset of all commodities,
$SHENE_{c,a}$ is the energy consumption share of energy commodity c in production activity a,
$PQ_{c,a}$ is the price of commodity c in production activity a (monetary/physical unit),
$\delta_{c,a}^{en}$ and β^{el} are parameters for logit selection, and
ae_c is the fuel-wise energy preference change parameter of commodity c.

For the stand-alone model, βae_a^B and $\delta_{c,a}$ are calibrated by base year information. Autonomous Energy Efficiency Improvement (AEEI) in the stand-alone model at_a is one of the critical parameters determining energy consumption. We adopted a uniform AEEI across energy end-use sectors for each year, which is associated with GDP growth. For the years that assumes more than 1% of GDP annual growth, AEEI change rate is 1%, and half of the annual GDP growth rates is assumed for the other years. The fuel-wise energy preference change parameter ae_c is set annually to 1%, 0.5% and -0.5% for electricity, gas and coal respectively, which represent fuel shift from conventional solid and liquids to gas and electricity. They are arbitrarily assumed and their validity should be interpreted within the baseline scenario's perspective.

With respect to the integrated model, the $SHENE_{c,a}$ and $QENE_a^B$ are fixed based on the AIM/Enduse model results and endogenise $\delta_{c,a}^{en}$ and at_a.

Then, we can derive the input coefficients of capital in the baseline scenarios after obtaining the simulation results of the baseline scenarios as shown in Equation (6). For the mitigation scenarios, the derived input coefficients of the primary factors shown above are used to estimate primary factor inputs. For the capital inputs of the energy end-use sector, additional investment costs (associated with mitigation scenarios) relative to baseline scenarios, which are given by the AIM/Enduse model, have been added to the baseline inputs, as shown in Equation (7).

$$ifa_{f,a}^B = QF_{f,a}^B / QA_a^B \quad f \in Fcap \tag{6}$$

$$QF_{f,a}^M = ifa_{f,a}^B * QA_a^M + AddInv_{f,a} \quad f \in Fcap \tag{7}$$

where
$f \in Fcap$ is a set of capital and a subset of primary factors
ifa_a^B is an input coefficient of primary factor f and sector a in the baseline scenarios.
$AddInv_{f,a}$ is the additional investment cost associated with mitigation costs provided by AIM/Enduse,
$QF_{f,a}^M$ is the primary factor input of factor f and sector a in the mitigation scenarios (f = capital or labour) (monetary unit), and
QA_a^M is an output of sector a in the mitigation scenarios (monetary unit).

2) Power generation in CGE model

The total consumption of electricity is determined by the demand side representation shown in the previous subsection (*QENE* and *SHEHE*, and the latter for household). Then, the power generation is determined based on logit sharing which has already been mentioned above, as below.

$$SHAC_{c,a} = \frac{\delta^{el}_{c,ap} \cdot PXAC_{c,a}^{-\beta^{el}}}{\sum_{ap \in AEly} \delta^{el}_{c,ap} \cdot PXAC_{c,ap}^{-\beta^{el}}} \quad c \in CEly, a \in AEly \tag{8}$$

where $a \in AEly$ is a subset of production activity and electricity production activity (e.g. coal, solar PV and so on),
$c \in CEly$ is a subset of commodity and electricity,
$SHAC_{c,a}$ is the electricity generation share of production activity a,
$PXAC_{c,a}$ is the price of commodity *c* produced by production activity a (monetary/physical unit),
$\delta^{el}_{c,a}$ and β^{el} are parameters for the logit selection of power general technologies.

For the stand-alone model, $\delta^{el}_{c,a}$ is calibrated using base year information. The integrated model fixes the $SHAC_{c,a}$ as the AIM/Enduse model results and endogenises $\delta^{el}_{c,a}$. This treatment is the same between the baseline and the mitigation scenarios. As the absolute amount of power generation is determined by the demand side, here we specify only the share. The transmission losses are also considered. Battery capacity is input as an absolute amount.

3) Household consumption in CGE model

Household consumption is formulated using a LES function and further nested in a CES for energy and other manufacturing goods. The function is derived from a function originally defining how spending on individual commodities has a linear relation with total consumption spending, based on the assumption that each household maximises a Stone–Geary utility function subject to consumption expenditure constraints. The parameters of the LES function other than food were calibrated based on income elasticity values (Nganou, 2005)[24]. The income elasticity of food demand for each region and commodity was from Bruinsma (2010)[25].

Then, total energy consumption is finally split out into fuel-wise consumption using a logit function

$$QCH_{ch} = \mu_{ch} + \theta_{ch} \cdot \left(\frac{EH}{PCH_{ch}} - \sum_{chp \in CH} \mu_{chp}\right) \quad ch \in CH \tag{9}$$

$$QCH_{ch} = \alpha h_{ch} \left(\beta h_{ch} \cdot QHE_{ch}^{-\rho h_{ch}} + (1 - \beta h_{ch}) \cdot QHM_{ch}^{-\rho h_{cp}}\right)^{\frac{1}{-\rho h_{ch}}} \tag{10}$$

$$SHHENE_{c,ch} = \frac{ae_c \cdot \delta^{h}_{c,ch} \cdot PQ_{c,h}^{-\beta^h}}{\sum_{cp \in CENE} ae_{cp} \cdot \delta^{h}_{cp,ch} \cdot PQ_{cp,h}^{-\beta^h}} \quad c \in CENE, ch \in CHE \tag{11}$$

$$QH_c = \begin{cases} \sum_{ch \in CHE} SHHENE_{c,ch} \cdot QHE_{ch} & c \in CENE \\ \sum_{(c,ch) \in mapCCH} QCH_{ch} & c \in CNENE \\ \sum_{(c,ch) \in mapCCH} QHM_{ch} & c \in COMF \end{cases} \tag{12}$$

where
$ch \in CH$ is a set of household consumption goods of which mappings with goods *c* are shown in Supplementary Table 11
$ch \in CHE$ is a set of energy related household consumption goods (car usage and other energy related consumption)
$(c, ch) \in mapCCH$ is a mapping from household consumption goods *ch* to general goods *c*. shown in Supplementary Table 11,
PCH_{ch} are the quantity and price of household consumption goods ch (relative ratio to base year),
QCH_{ch} are the quantity and price of household consumption goods ch (monetary unit but energy is accounted as physical unit),
$SHHENE_{c,ch}$ is the share of energy fuel c of household consumption goods *ch*,
QHE_{ch} and QHM_{ch} are the quantity of energy and other manufacturing goods, respectively, for household consumption goods *ch* (car usage and other energy related consumption; physical unit),
θ_{ch} and $\mu_{ch}$are LES function parameters which are calibrated recursively,
αh_{ch} is the scale parameter of the CES function for energy and other manufacturing goods aggregates,
βh_{ch} is the share parameter of the CES function for energy and other manufacturing goods aggregates,
ρh_{ch} is the exponent parameter of the CES function for energy and other manufacturing goods aggregates,
$\delta^{h}_{c,ch}$ and β^h are parameters for logit selection of household energy fuel.

Household LES function parameters are updated recursively based on income elasticity. Electricity and biofuel used in transport, are not accounted in the base year social accounting matrix and, thus, we introduce the initial parameters for $\delta^{h}_{c,ch}$ and $\delta^{en}_{c,a}$ by calibrating 0.1% of the share in each energy consumption in 2015 and 2020. They are then updated afterwards to one third of the value of petroleum products in 40 years. They are same as used in the SSPs quantification. For the integrated model, the parameters βh_{ch} and ρh_{ch} were determined endogenously based on the energy consumption and investment needs for other manufacturing goods computed by AIM/Enduse.

Supplementary Note 3. Consistency check across models

We confirm how the outputs of two models change over iterations and reach convergence. Supplementary Table 12 shows the degree of coincidence for each pair of AIM/Enduse and AIM/CGE datasets. An indicator shown below is adopted as the indicator. i, t and s are sets of variables of model (e.g. energy demand), years and scenarios respectively. $X_{i,t,s}$ and $Y_{i,t,s}$ are AIM/CGE and AIM/Enduse outputs respectively.

$$ErrI_{i,s} = \sqrt{\frac{\sum_t \left(X_{t,i,s} - Y_{t,i,s}\right)^2}{\left[\sum_t \left(\frac{X_{t,i,s} + Y_{t,i,s}}{2}\right)\right]^2}}$$

CGE results drastically changed from the standalone version to the coupled version, and the discrepancy with AIM/Enduse falls substantially (End1_CGE1 to End1_CGE2 in Supplementary Figure 5). The differences in these improvements are nearly stable in the second iteration run, wherein AIM/Enduse incorporated energy service demand changes given by AIM/CGE and AIM/CGE further input revised AIM/Enduse data (End1_CGE2 to End2_CGE3 in Supplementary Figure 5). We run five iterations and the results indicate that the second iteration sufficiently converges the variables (see Supplementary Figure 9 to Supplementary Figure 14). This result may be due to energy service demand changes produced by AIM/CGE, which fed into AIM/Enduse, being small in the second and third iterations. Here, we can estimate the value-added and household consumption changes (differences between CGE2 and CGE3 in Supplementary Figure 5). Consequently, the third CGE run showed few changes from the second run.

Overall, the errors are less than 20%, but there are some exceptions. Final energy in the residential and service sectors and electricity generation from oil and hydropower in the baseline scenarios exhibit relatively high discrepancies, which show relatively small improvements in the third iteration. Therefore, we classified two types of errors. First, some years have very small values in one or both models, causing the error rates of these two models to be large in some years. Oil-fired power generation is one such example, as shown in Supplementary Figure 16. The second type of error is mainly due to discrepancies in the base year information between the two models. The residential and service sectors' final energy consumption and hydropower explain the cause of these differences, as shown in Supplementary Figure 17. As indicated in the Methods section, we incorporated the change ratios from AIM/Enduse into AIM/CGE relative to the base year for final energy consumption, and thus trends after the base year for AIM/Enduse, which is 2010, are quite similar in the second AIM/CGE run. We further provide figures comparing energy-related data, Supplementary Figure 18 and Supplementary Figure 19, to explore the future evolution of energy systems.

Regarding the consistency of the AIM/Power and AIM/Enduse models, power generation appears to be almost the same at the end of the run (Supplementary Figure 20). Caution is still required, as other indicators such as capacity factor, may play a role here. That possibility is not necessarily true for the detailed technological contributions, but would hardly affect the overall trend because the main differences affect tiny power generation sources (e.g. the coal power capacity factor in mitigation scenarios).

4. Supplementary references

1. Shiraki H, Ashina S, Kameyama Y, Hashimoto S, Fujita T. Analysis of optimal locations for power stations and their impact on industrial symbiosis planning under transition toward low-carbon power sector in Japan. *Journal of Cleaner Production* 2016, **114:** 81-94.

2. International Institute for Applied Systems Analysis, (IIASA). IAMC AR5 scenario database. 2015 [cited]Available from: https://secure.iiasa.ac.at/web-apps/ene/AR5DB/dsd?Action=htmlpage&page=about#intro

3. Abrell J, Rausch S. Cross-country electricity trade, renewable energy and European transmission infrastructure policy. *Journal of Environmental Economics and Management* 2016, **79:** 87-113.

4. Andersen KS, Termansen LB, Gargiulo M, Ó Gallachóirc BP. Bridging the gap using energy services: Demonstrating a novel framework for soft linking top-down and bottom-up models. *Energy* 2019, **169:** 277-293.

5. Arndt C, Davies R, Gabriel S, Makrelov K, Merven B, Hartley F*, et al.* A sequential approach to integrated energy modeling in South Africa. *Applied Energy* 2016, **161:** 591-599.

6. Böhringer C, Rutherford TF. Combining bottom-up and top-down. *Energy Economics* 2008, **30**(2)**:** 574-596.

7. Simões S, Seixas J, Van Regemorter D, Ferreira F. Top-down and bottom-up modelling to support low-carbon scenarios: climate policy implications AU - Fortes, Patricia. *Climate Policy* 2013, **13**(3)**:** 285-304.

8. Helgesen PI, Tomasgard A. From linking to integration of energy system models and computational general equilibrium models – Effects on equilibria and convergence. *Energy* 2018, **159:** 1218-1233.

9. Hwang W-S, Lee J-D. A CGE analysis for quantitative evaluation of electricity market changes. *Energy Policy* 2015, **83:** 69-81.

10. Krook-Riekkola A, Berg C, Ahlgren EO, Söderholm P. Challenges in top-down and bottom-up soft-linking: Lessons from linking a Swedish energy system model with a CGE model. *Energy* 2017, **141:** 803-817.

11. Lanzi E, Chateau J, Dellink R. Alternative approaches for levelling carbon prices in a world with fragmented carbon markets. *Energy Economics* 2012, **34:** S240-S250.

12. Drouet L, Haurie A, Labriet M, Thalmann P, Vielle M, Viguier L. A Coupled Bottom-Up/Top-Down Model for GHG Abatement Scenarios in the Swiss Housing Sector. In: Loulou R, Waaub J-P, Zaccour G (eds). *Energy and Environment*. Springer US, 2005, pp 27-61.

13. Sue Wing I. The synthesis of bottom-up and top-down approaches to climate policy modeling: Electric power technology detail in a social accounting framework. *Energy Economics* 2008, **30**(2)**:** 547-573.

14. Tapia-Ahumada K, Octaviano C, Rausch S, Pérez-Arriaga I. Modeling intermittent renewable electricity technologies in general equilibrium models. *Economic Modelling* 2015, **51:** 242-262.

15. Tuladhar SD, Yuan M, Bernstein P, Montgomery WD, Smith A. A top–down bottom–up modeling approach to climate change policy analysis. *Energy Economics* 2009, **31:** S223-S234.

16. Vandyck T, Keramidas K, Saveyn B, Kitous A, Vrontisi Z. A global stocktake of the Paris pledges: Implications for energy systems and economy. *Global Environmental Change* 2016, **41**(Supplement C)**:** 46-63.

17. Waisman H, Guivarch C, Grazi F, Hourcade JC. The Imaclim-R model: infrastructures, technical inertia and the costs of low carbon futures under imperfect foresight. *Climatic Change* 2012, **114**(1)**:** 101-120.

18. Oshiro K, Masui T. Diffusion of low emission vehicles and their impact on CO2 emission reduction in Japan. *Energy Policy* 2015, **81:** 215-225.

19. van der Werf E. Production functions for climate policy modeling: An empirical analysis. *Energy Economics* 2008, **30**(6)**:** 2964-2979.

20. McFadden D. Econometric models of probabilistic choice. *Structural analysis of discrete data with econometric applications* 1981, **198272**.

21. Brenkert AL, Smith SJ, Kim SH, Pitcher HM. Model Documentation for the MiniCAM. PNNL; 2003.

22. de Vries BJM, van Vuuren DP, den Elzen MGJ, Janssen MA. The Targets IMage Energy Regional (TIMER) model Technical Documentation. Department of International Environmental Assessment
National Institute of Public Health and the Environment (RIVM); 2001.

23. Clarke JF, Edmonds JA. Modelling energy technologies in a competitive market. *Energy Economics* 1993, **15**(2)**:** 123-129.

24. Nganou J-P. Estimation of the parameters of a linear expenditure system (LES) demand; 2005.

25. Bruinsma J. The resource outlook to 2050: by how much do land, water and crop yields need to increase by 2050?, Expert meeting on how to feed the world in 2050; 2010.
