# Supplementary material for: Energy transformation cost for the Japanese mid-century strategy
Source: Nat Commun. 2019 Oct 18;10:4737. doi: 10.1038/s41467-019-12730-4 (PMC6800421; doi:10.1038/s41467-019-12730-4)
Supplement: Supplementary file 3 — Reporting Summary [file 41467_2019_12730_MOESM3_ESM.pdf]

nature research

Corresponding author(s): Shinichiro Fujimori

Last updated by author(s): 2019-9-21

Reporting Summary

Nature Research wishes to improve the reproducibility of the work that we publish. This form provides structure for consistency and transparency in reporting. For further information on Nature Research policies, see Authors & Referees and the Editorial Policy Checklist.

Statistics

For all statistical analyses, confirm that the following items are present in the figure legend, table legend, main text, or Methods section.

n/a	Confirmed	
☐	☒	The exact sample size (*n*) for each experimental group/condition, given as a discrete number and unit of measurement
☒	☐	A statement on whether measurements were taken from distinct samples or whether the same sample was measured repeatedly
☐	☒	The statistical test(s) used AND whether they are one- or two-sided<br>*Only common tests should be described solely by name; describe more complex techniques in the Methods section.*
☒	☐	A description of all covariates tested
☐	☒	A description of any assumptions or corrections, such as tests of normality and adjustment for multiple comparisons
☐	☒	A full description of the statistical parameters including central tendency (e.g. means) or other basic estimates (e.g. regression coefficient) AND variation (e.g. standard deviation) or associated estimates of uncertainty (e.g. confidence intervals)
☒	☐	For null hypothesis testing, the test statistic (e.g. *F*, *t*, *r*) with confidence intervals, effect sizes, degrees of freedom and *P* value noted<br>*Give P values as exact values whenever suitable.*
☒	☐	For Bayesian analysis, information on the choice of priors and Markov chain Monte Carlo settings
☒	☐	For hierarchical and complex designs, identification of the appropriate level for tests and full reporting of outcomes
☒	☐	Estimates of effect sizes (e.g. Cohen's *d*, Pearson's *r*), indicating how they were calculated

Our web collection on statistics for biologists contains articles on many of the points above.

Software and code

Policy information about availability of computer code

Data collection	The scenario data is generated by an economic (AIM/CGE), an energy system(AIM/Enduse) and a power dispatch (AIM/Power) model. The current code base of the AIM/Enduse, AIM/CGE and AIM/POWER developed over more than two decades at Kyoto University, Shiga Prefecture University and National Institute for Environmental Studies. AIM/Enduse is available at AIM website (http://www-iam.nies.go.jp/aim/data_tools/index.html#enduse) and others are not available in a publicly shareable version. The code will continue to be developed and hosted by Kyoto University, Department of Environmental Engineering (http://www.athehost.env.kyoto-u.ac.jp/). Requests for code should be addressed to Shinichiro Fujimori.
Data analysis	We used R code for data analysis and visualization which is open to the public

For manuscripts utilizing custom algorithms or software that are central to the research but not yet described in published literature, software must be made available to editors/reviewers. We strongly encourage code deposition in a community repository (e.g. GitHub). See the Nature Research guidelines for submitting code & software for further information.

Data

Policy information about availability of data

All manuscripts must include a data availability statement. This statement should provide the following information, where applicable:

- Accession codes, unique identifiers, or web links for publicly available datasets
- A list of figures that have associated raw data
- A description of any restrictions on data availability

Scenario data is accessible online via HARVARD Dataverse (https://doi.org/10.7910/DVN/QE6ERU). The data which is derived from the original scenario database shown as figures but not in the above database is available upon requests.

Field-specific reporting

Please select the one below that is the best fit for your research. If you are not sure, read the appropriate sections before making your selection.

☐ Life sciences ☐ Behavioural & social sciences ☒ Ecological, evolutionary & environmental sciences

For a reference copy of the document with all sections, see nature.com/documents/nr-reporting-summary-flat.pdf

Ecological, evolutionary & environmental sciences study design

All studies must disclose on these points even when the disclosure is negative.

Study description	The future scenarios are estimated by a computer simulation model. Then, the simulation results are assessed.
Research sample	Japan and single model results are used.
Sampling strategy	There is no sampling strategy.
Data collection	The data is recorded by authors. The economic, energy system, and power model's outputs are handled by SF, KO and HS respectively.
Timing and spatial scale	The year from 2010 to 2050 annually and Japan.
Data exclusions	None
Reproducibility	The scenario data can be generated by the computer simulation model. If it is required for the review purpose, we could make it available for the reviewers.
Randomization	The scenario data are not random sampling data which does not have any blinding method.
Blinding	The scenario data are not random sampling data which does not have any blinding method.

Did the study involve field work? ☐ Yes ☒ No

Reporting for specific materials, systems and methods

We require information from authors about some types of materials, experimental systems and methods used in many studies. Here, indicate whether each material, system or method listed is relevant to your study. If you are not sure if a list item applies to your research, read the appropriate section before selecting a response.

Materials & experimental systems

n/a	Involved in the study	
☒	☐	Antibodies
☒	☐	Eukaryotic cell lines
☒	☐	Palaeontology
☒	☐	Animals and other organisms
☒	☐	Human research participants
☒	☐	Clinical data

Methods

n/a	Involved in the study	
☒	☐	ChIP-seq
☒	☐	Flow cytometry
☒	☐	MRI-based neuroimaging
